# Supplementary material for: Vitamin D Modified DSS-Induced Colitis in Mice via STING Signaling Pathway
Source: Biology (Basel). 2025 Jun 18;14(6):715. doi: 10.3390/biology14060715 (PMC12190092; doi:10.3390/biology14060715)
Supplement: Supplementary file 1 [file biology-14-00715-s001.zip › Supplementary Materials S1: 16s-report of gut microbiota/Groups/diff_analysis/RF/FAPROTAX_Groups.RFimportance.pdf]

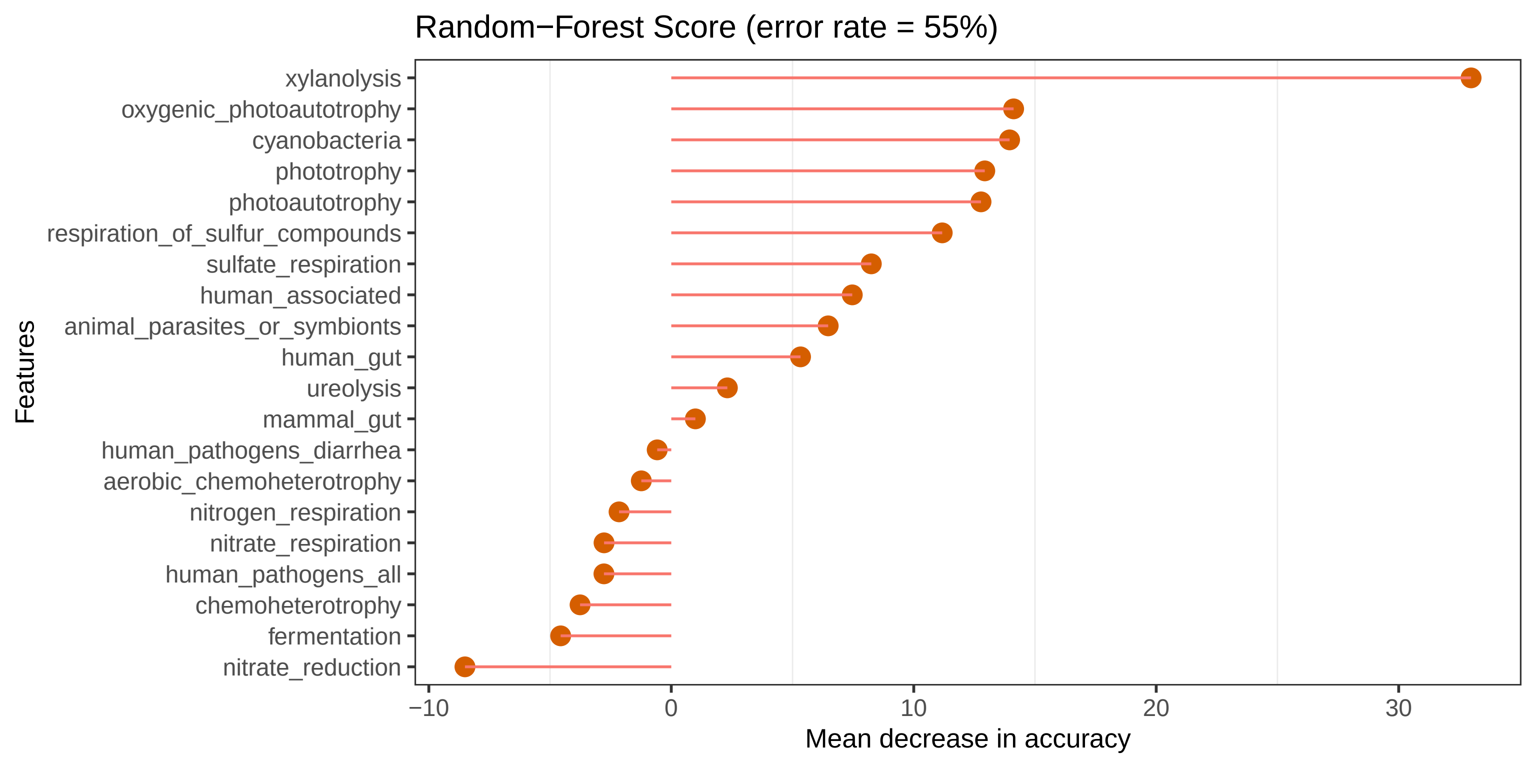

Random−Forest Score (error rate = 55%)
xylanolysis
oxygenic_photoautotrophy
cyanobacteria
phototrophy
photoautotrophy
respiration_of_sulfur_compounds
sulfate_respiration
human_associated
animal_parasites_or_symbionts
human_gut
ureolysis
mammal_gut
human_pathogens_diarrhea
aerobic_chemoheterotrophy
nitrogen_respiration
nitrate_respiration
human_pathogens_all
chemoheterotrophy
fermentation
nitrate_reduction
Features
−10
0
10
20
30
Mean decrease in accuracy
